# Implementation of facemask sampling for the detection of infectious individuals with SARS-CoV-2 in high stakes clinical examinations – a feasibility study

**DOI:** 10.1016/j.fhj.2024.100175

**Published:** 2024-09-05

**Authors:** Daniel Pan, Caroline Williams, Jonathan Decker, Eve Fletcher, Natalia Grolmusova, Paul W. Bird, Christopher A. Martin, Joshua Nazareth, Latif Rahman, Kate O'Kelly, Rakesh Panchal, Irfana Musa, Harshil Dhutia, Shirley Sze, Manish Pareek, Michael R. Barer

**Affiliations:** a Development Centre for Population Health, University of Leicester, UK; b Department of Respiratory Sciences, University of Leicester, UK; c Department of Infectious Diseases and HIV Medicine, University Hospitals of Leicester NHS Trust, UK; d NIHR Leicester Biomedical Research Centre, UK; e Li Ka Shing Centre for Health Information and Discovery, University of Oxford, UK; f WHO Collaborating Centre for Infectious Disease Epidemiology and Control, School of Public Health, Li Ka Sing Faculty of Medicine, University of Hong Kong, Hong Kong, China; g Department of Microbiology, University Hospitals of Leicester NHS Trust, UK; h Department of Acute Medicine, University Hospitals of Leicester NHS Trust, UK; i Department of Geriatric Medicine, University Hospitals of Leicester NHS Trust, UK; j Department of Respiratory Medicine, University Hospitals of Leicester NHS Trust, UK; k Department of Cardiology, University Hospitals of Leicester NHS Trust, UK; l Department of Cardiovascular Sciences, University of Leicester, UK

**Keywords:** SARS-CoV-2, Transmission, PACES, Examination, Acceptability

## Abstract

**Introduction:**

SARS-CoV-2 may transmit across vaccinated cohorts during practical clinical examinations. We sought to assess the feasibility of facemask sampling (FMS) to identify individuals emitting SARS-CoV-2 during a mock PACES exam.

**Methods:**

In May 2022 we recruited participants from a mock PACES examination in Leicester, UK. Following a negative lateral flow test assay, all participants wore modified facemasks able to capture exhaled virus during the assessment (FMS). A concomitant upper respiratory tract sample (URTS) was provided prior to FMS. Exposed facemasks were processed by removal and dissolution of sampling matrices fixed within the mask and cycle thresholds values quantified by RT-qPCR. Participants were asked to grade statements regarding the comfort, effort, ethics and communication when providing FMS; laboratory technicians were asked to grade key statements surrounding suitability of samples for processing.

**Results:**

34 participants provided concomitant URTS and FMS during the examination. One participant was positive for SARS-CoV-2, with a cycle threshold value of 22.5 on URTS, but negative (no viral RNA detected) on FMS; no transmission to others was identified from this individual. Participants responded positively to statements regarding FMS describing all four domains; however, 69% of participants felt that a positive result from FMS alone was insufficient for diagnosis and that further tests were required. All but one FMS sample was suitable for processing.

**Discussion:**

FMS during PACES exams are acceptable among participants and samples provided are suitable for processing. Our results demonstrate feasibility of FMS within practical examination settings and support the further assessment of FMS as a scalable tool that can be compared with URTS to identify those who are infectious.

## Introduction

The COVID-19 pandemic has affected training and assessment guidelines for all medical trainees. In the UK, doctors who wish to enter higher specialist training in internal medicine are required to complete an examination known as the Membership of the Royal College of Physicians of the United Kingdom (MRCP(UK)) Diploma.^1^ The final part is a clinical assessment (PACES) that involves eight real patient encounters for each candidate, during which core clinical skills are assessed. Here, volunteer patients are often in the same rooms as examiners for prolonged periods of time while being exposed to several examination candidates, making them especially vulnerable to infection even if they have been fully vaccinated.^2^

To mitigate transmission, PACES candidates are asked to declare that they have had a negative lateral flow assay (LFA) rapid antigen test within 48 h of their examination.^3^ Candidates are also required to fill in a screening health questionnaire in advance of examination attendance and wear appropriate personal protective equipment (including facemasks). Appropriate physical distancing is also implemented, with patients who do not require physical examination interviewed by candidates in separate rooms. However, emerging data suggest that significant proportions of infectious individuals are missed with lateral flow tests, especially if they are fully vaccinated, early on in infection, or asymptomatic.^4^, ^5^, ^6^

We have previously shown that the capture of exhaled SAR-CoV-2 RNA by facemask sampling (FMS) is strongly associated with household transmission and can be positive in symptomatic patients with a concomitantly PCR-negative upper respiratory tract sample (URTS).^7^ To enable transmission, SARS-CoV-2 must be emitted from the respiratory tract. FMS offers particular advantages for assessment of exhaled virus output over prolonged sampling periods, such as its non-invasive nature, no requirement for trained staff to collect the sample and buffer-free storage and transport for processing.^8^ Other advantages include routine use of facemasks within healthcare and other settings; as such, FMS can serve as both an identifying test for those who are breathing out high quantities of virus, as well as source control within such these settings. For instance, over the last few years, the use of facemasks has been compulsory for PACES examination candidates.

In this study, we aimed to evaluate the experience of examination candidates who took part in a mock PACES examination while undertaking FMS. We report the acceptability of FMS within this environment and the suitability of the samples provided for processing. Our findings have implications for the potential use of FMS in high-stake examination settings – for both the reduction of airborne transmission, and also to identify those who may (or may not) be infectious, with minimal disruption to examination organisers and candidates.

## Methods

### Study settings

We enrolled healthcare workers (HCWs) who took part in the Leicester mock PACES examination in May 2022. This included examination candidates, examiners, observers and volunteers who helped with the running of the exam. Eight patients took part in the mock PACES but were not recruited into the study, since PACES guidance exempts patients from wearing a mask due to their chronic health conditions. All participants (including patients) must report a negative LFA 48 h prior to coming to the mock exam. The study has ethical approval from West Midlands Research Ethics Committee (REC Reference 20/WM/0153). All participants gave written, informed consent prior to any study procedures.

### Mock PACES exam

The mock PACES exam followed standards set for the real exam by the Royal College of Physicians.^9^ In brief, candidates are marked at five clinical stations, consisting of the assessment of eight patients, with each station assessed by two independent examiners. Patients and examiners remain in their respective rooms for the whole duration of the exam, while candidates are guided by helpers to different rooms for each station. Candidates start at any one of the stations and move to the next every 20 min until they have completed the cycle. There is a 5-min period between each station when the candidate waits outside the room of their next station.

### Sampling procedure

Our sampling methods have been described in detail previously.^7^^,^^8^ All participants in the mock PACES (excluding volunteer patients) provided a single FMS and URTS on the day of the mock exam. An URTS was taken to compare with URTS results should any participant test positive. An URTS was provided prior to the start of the exam; each participant then wore a duckbill surgical mask (Integrity 600-3004) containing two 1 × 9 cm 3D printed polyvinyl-alcohol (PVA) sampling matrix strips, placed horizontally across the inside of the mask for the whole duration of the mock examination (lasting 125 min). One study investigator (PWB) ensured that all participants wore the mask properly (with no breaks) during the course of the exam. FMS were collected from each participant following the end of the mock exam, as candidates were receiving feedback from examiners.

Exposed samples were transported to the laboratory immediately after the examination was complete and processed on the same day. If any participants did turn out to be positive on URTS or FMS, they would be informed within 24 h and asked to provide five further consecutive URTS and FMS samples in the 5 days following the exam. Other participants of the mock PACES examination as well as patients were then informed that one or more examination participants tested positive during the examination and to inform the study investigators should they become symptomatic. Patients were provided with sufficient supplies of LFAs and asked to test daily for 7 days following the examination, to detect any transmission events early. Patients who took part in the clinical examinations did not undergo testing, as per standard PACES examination protocols.

### Sampling processing and controls

Detailed description is provided in our previous publications.^7^^,^^8^ In brief, for FMS processing, two PVA strips were dissolved in a mixture of molecular-grade water and QIAamp ACL buffer and underwent RNA extraction using the QIAampl Circulating Nucleic Acid Kit (Qiagen, Cat 55114). For URTS, the sampled material was first eluted from the swab head into water by vortexing then RNA extracted using RNeasy mini kits (Qiagen, Cat 74104). For both sample types, target RNA was detected and quantified using the QuantiNova Probe RT-qPCR Kit (Qiagen, Cat 208356) and a Rotor-Gene Q thermocycler (Qiagen, Cat 9001590). Quantification results were normalised to per sampling strip for FMS, and to per 100 μl of swab eluate for URTS. Sample positivity was determined with assays directed to the E gene. Any positive samples were quantified for genome copy number in a single E gene-directed RT-qPCR run (see previous work for standard curve).

### Demographic data, acceptability of FMS and assessment of sampling quality

We collected data on age, gender, grade and ethnicity of participants. Following the end of the mock examination, we also asked participants to grade key statements regarding their experiences of using FMS, based on the following themes: comfort while wearing the mask; effort required to wear the mask; ethical implications of wearing a facemask during an examination; difficulties with communication while wearing a facemask; and beliefs surrounding COVID-19 transmission. Grading was with a five-point Likert scale; 1: strongly disagree; 2: disagree; 3: neutral; 4: agree; 5: strongly agree. Multiple statements, positively and negatively framed, were used to explore the same themes for reporting reliability. Similarly, two laboratory technicians (EF and JD) who received the FMS were asked to grade key statements surrounding the suitability of the samples for processing on a five-point Likert scale. The final score given to the FMS samples was agreed by both laboratory technicians.

### Statistical analysis

Continuous variables are expressed as median and interquartile range (IQR). Categorical variables are displayed as numbers and percentages (%). Pearson's chi-squared test and Fisher's exact row test were used to compare categorical variables between groups. Student's *t*-test and Kruskal–Wallis were used to compare continuous variables between groups depending on the normality of distribution. Bar charts were used to illustrate data from the Likert scale. Data were analysed using GraphPad Prism (version 9), Excel (Microsoft 2010) and STATA (version 16.1). All tests were two-tailed and *p* values <0.05 were regarded as significant.

## Results

A total of 34 HCWs participated in the mock PACES and consented to providing FMS and URTS for the duration of the examination; their demographics are shown in Table 1. Most participants were male (65%). 15 (44%) were junior doctors who took part in the mock exam; 13 (38%) were senior registrars or consultants who acted as examiners; six (18%) were foundation year doctors or medical students that helped to ensure the mock exam went smoothly. Half of the cohort was South Asian in ethnic origin; around a third (37%) were Caucasian.

**Table 1:** Baseline characteristics of the cohort.

Category	*N* = 34
Male sex (%total)	22 (65%)
Age median (IQR)	30 (28–32)
*Grade*	
Foundation/med student	6 (18%)
Senior house officer	15 (44%)
Specialist registrar	6 (18%)
Consultant	7 (21%)
*Race*	
Caucasian	9 (27%)
East Asian	4 (12%)
Middle Eastern and Black	4 (12%)
South Asian	17 (50%)

**Fig. 1:**
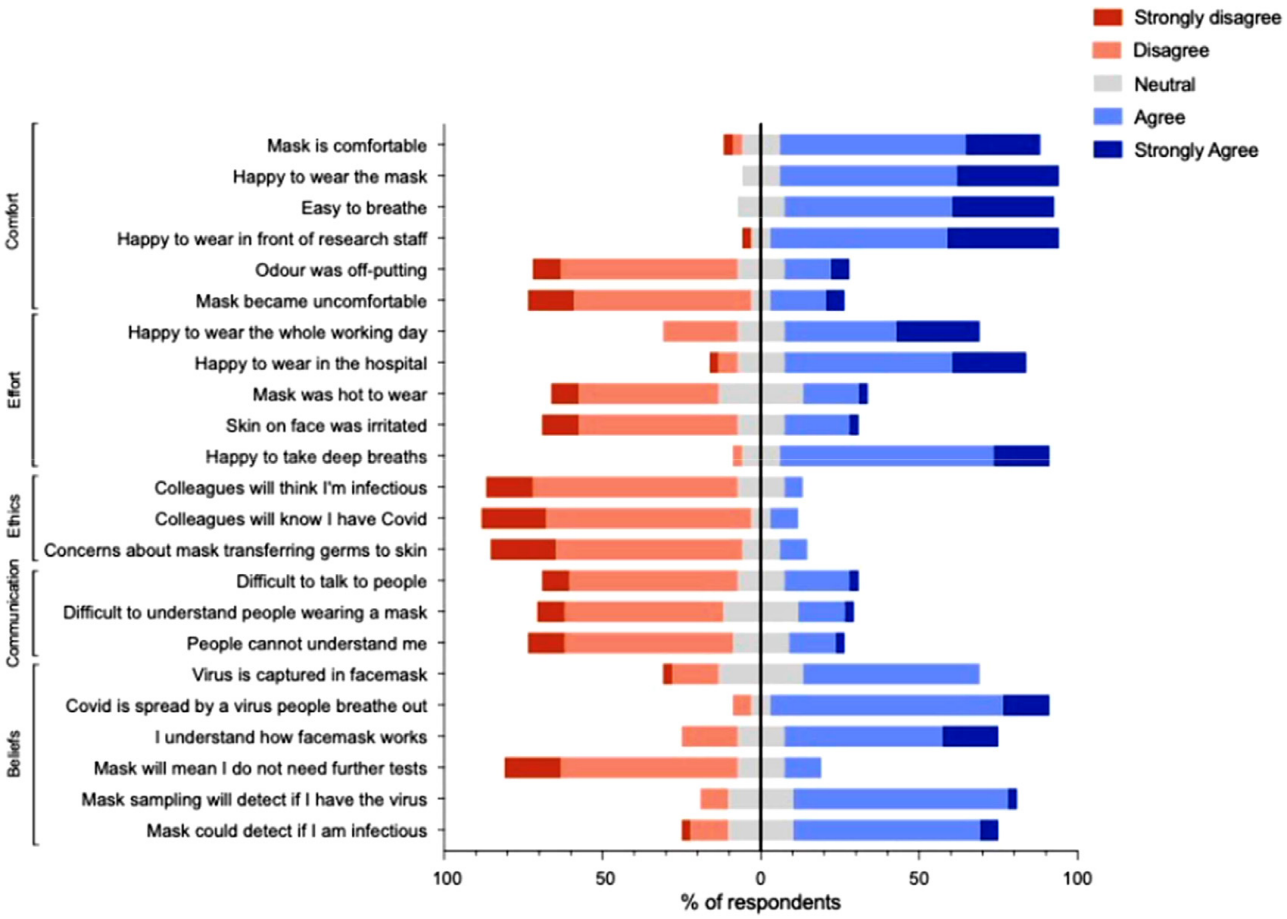
Degree of agreement to statements regarding comfort, effort, ethics, communication and beliefs surrounding facemask sampling.

The mock exam ran without any significant delays from FMS. Fig. 1 and appendix 1 show Likert responses from participants regarding key themes of FMS. In general, FMS was positively received, with most participants finding the mask provided for FMS to be comfortable to wear for the duration of the exam, required minimum effort to use and ethically sound. Most also agreed that communication was not difficult while wearing the facemask and understood that FMS was being performed for the detection of SARS-CoV-2. The majority felt that a positive result from FMS alone was insufficient for diagnosis and that further tests were required. No differences in responses were detected in the responses by gender, age, grade or ethnic group (Appendix 2).

**Fig. 2:**
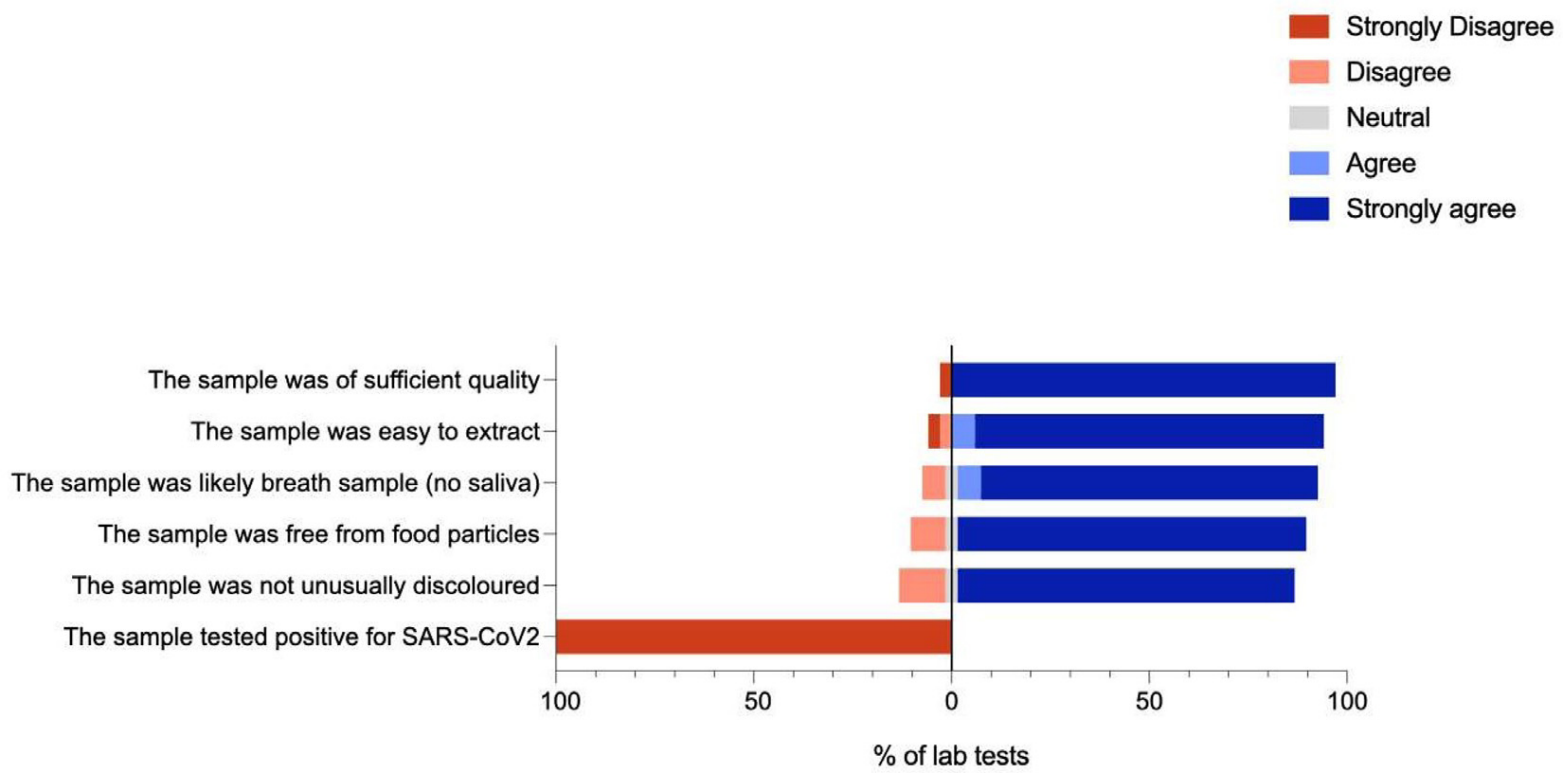
Degree of agreement to statements regarding suitability of samples for processing.

Fig. 2 reports the suitability of FMS samples from the mock exam for processing, as reported by laboratory technicians; all samples were suitable for RNA extraction. Of 34 samples processed, four (12%) samples were found to be unusually discoloured; three (8%) separate samples were found to contain food particles; these samples however did not appear to affect PCR processing. No FMS was positive; however, one participant was identified to be positive for SARS-CoV-2 on URTS PCR, with a cycle threshold value of 22.5 for E gene. The participant was contacted for contact tracing and reported that he had recently tested positive for SARS-CoV-2 on PCR from URTS 3 weeks prior to the study but was LFA negative on the day of the exam. All remaining participants and patients were informed that someone had tested positive on one of the samples during the exam; none reported developing any symptoms suspicious of COVID-19 in the 2 weeks following the study. Subsequent FMS, URTS PCR and LFA samples on 5 consecutive days following the mock exam from the participant who initially tested positive on URTS were negative for SARS-CoV-2.

## Discussion

SARS-CoV-2 remains highly transmissible within vaccinated populations.^10^ FMS offers the advantages of simultaneously identifying individuals who are exhaling virus as an examination is taking place while decreasing the risk of transmission to others through a physical barrier over the nose and mouth.^11^, ^12^, ^13^, ^14^ Facemasks are also wearable devices, allowing for convenience when sampling for prolonged period and already available commercially at low cost. In this study, we found that it is possible to successfully implement FMS during the course of a high-stakes mock practical examination. We show that most participants were amenable to FMS and that samples provided were suitable for processing.

Older patients, as well as those with multiple comorbidities (such as immunosuppression) and frailty, are at higher risk of developing severe disease once infected with SARS-CoV-2, even with pre-existing immunity through previous infection or vaccination.^2^^,^^15^^,^^16^ Such patients are commonly asked to take part in both undergraduate and postgraduate clinical examinations since clinical signs of chronic disease tend to manifest only in its most advanced stages. An analysis of the most common cases to come up in PACES stations include interstitial lung disease, transplanted kidney, prosthetic heart valves, Parkinson's disease and rheumatoid arthritis; these cohorts are all at risk of adverse outcomes from COVID-19, or have suboptimal immune protection from vaccination or previous infection.^16^, ^17^, ^18^, ^19^, ^20^ The Royal College of Physicians and other organisations that organise practical clinical examinations have a duty to protect these patients, prevent outbreaks within examination settings as well as ensure that candidates and examiners can conduct the examination as smoothly as possible.^21^

Our study illustrates the possibility of using FMS as one part of the solution for infection prevention in high stakes examination settings, allowing for detection of individuals who are actively emitting virus while causing as little discomfort or disruption to the examination process. Incubation periods for SARS-CoV-2 and other respiratory viruses are usually 3–6 days. PCR results from FMS can be performed within 24 h of the event, taking into account time to transport to laboratory. Therefore, vulnerable contacts of a participant who is emitting virus captured by FMS can be informed within 24 h following the exam, so that they can test themselves in a timely fashion and seek medical support if necessary. This is particularly relevant for SARS-CoV-2, where there is high variability in the time to onset of symptoms following infection. In addition, the commencement of antivirals for COVID-19 in vulnerable individuals (often fitting those who participate in PACES examinations) are most effective the earlier they are started during the natural history of infection, often prior to symptom onset.

We have previously demonstrated that FMS is a simple and effective method for the detection and quantification of exhaled SARS-CoV-2 in hospitalised patients with COVID-19, as well as patients in the community with milder symptoms. In these studies, we found that viral load on FMS was associated with both disease severity and the likelihood of onward household transmission, with the latter outcome more strongly associated with FMS than URTS.^7^^,^^8^ During early infection, high FMS viral loads can be detected in the context of low viral loads from URTS within individuals who are highly symptomatic and low viral loads on FMS have high negative predictive values for the absence of household transmission. FMS was also used in SARS-CoV-2 human challenge studies, where viral RNA was detected in four masks (22% of infected participants) before the onset of any reported symptoms and three participants were shown to have emitted virus into masks before their first RDT positive sample on URTS.^22^ Interestingly, while quantity of RNA on URTS associates with likelihood of finding replication-competent virus, as well as LFA positivity, LFAs are poor at identifying human-to-human transmission events, especially in screening studies, where many infected are asymptomatic. While there are many reasons for why this could be, one possibility is that not all virus within the upper respiratory tract would be breathed out into the atmosphere, especially if the participant is asymptomatic. FMS, on the other hand, captures virus that is directly breathed out by an index case, and may better represent virus that is exposed to vulnerable contacts. The current study would support this hypothesis; one participant who tested positive on URTS with a low cycle threshold (indicating high likelihood of replication-competent virus) was FMS negative and did not, as far as we know, transmit to anyone else in the examination. Should this be the case, then FMS may offer advantages in this specific examination setting by identifying those who are most infectious, not those who simply test positive for SARS-CoV-2. Therefore, in theory, some examination candidates would still be able to take the PACES examination if they had recently recovered from a respiratory viral illness, where they may continue to test positive on URTS, but are actually no longer infectious.

Our study was limited in size, but reflective of the number of participants in real-life practical clinical examinations. While previous FMS studies have been done in larger numbers of individuals, ours is the largest number of samples to have been collected within 125 min from the start of sampling, demonstrating potential for scalability and screening within enclosed settings.^7^^,^^8^^,^^23^, ^24^, ^25^, ^26^ Demonstration of feasibility within a routine examination setting in one centre would allow us to further develop the use of FMS for further studies within other larger enclosed settings, where respiratory virus transmission could be a problem, such as care homes. We also did not directly ask participants whether they preferred URTS or FMS as a diagnostic tool for COVID-19; many in our survey were not convinced that FMS alone is sufficient for SARS-CoV-2 diagnosis. Previous work has shown that signals on FMS differ from those on URTS – therefore while URTS may be more sensitive as a tool for the diagnosis of COVID-19, FMS may be more sensitive as a tool to measure individual infectiousness.^7^^,^^8^ Without the evidence presented here demonstrating ease of FMS implementation, larger studies comparing diagnostic sensitivity of FMS versus URTS (or standard infection prevention procedures within hospitals) would be difficult to justify. We did not perform viral culture on either FMS or URTS; viral culture is not routinely performed in NHS laboratories; is labour intensive; requires laboratory facilities with high biosafety levels (ie category 3 or biosafety level 3 facilities), and for which a cytopathic effect can take 3–6 days to be observed. Our aim was to assess feasibility of a tool that could identify infectious individuals rapidly and more accurately than current tests and have the potentially to be applied in routine clinical practice. Successful viral culture from FMS has been performed within other studies. Finally, all participants were HCWs who were used to wearing masks as part of everyday work; implementation of FMS may be more challenging in occupations where facemasks are not compulsory.

In conclusion, we successfully implemented the use of FMS within the context of a postgraduate clinical examination. FMS was well received by exam participants and has potential to be used for the detection of SARS-CoV-2 in other high-stakes clinical examination settings. Larger studies are required to see if this intervention can be performed at scale; to compare the diagnostic sensitivity of SARS-CoV-2 on FMS versus URTS, identification of those who are and are not infectious (versus LFA) as well as cost effectiveness against both URTS and LFA. Acceptability of FMS tests, used in isolation and with URTS and LFA, should also be assessed within larger cohorts. Additionally, other cohorts where FMS may be useful, such as HCWs and patients within routine care in hospitals and care homes, should also be assessed in future studies.

## CRediT authorship contribution statement

**Daniel Pan:** Writing – review & editing, Writing – original draft, Visualization, Validation, Software, Resources, Project administration, Methodology, Investigation, Funding acquisition, Formal analysis, Data curation, Conceptualization. **Caroline Williams:** Writing – review & editing, Conceptualization. **Jonathan Decker:** Writing – review & editing, Data curation, Conceptualization. **Eve Fletcher:** Writing – review & editing, Validation, Data curation. **Natalia Grolmusova:** Writing – review & editing, Writing – original draft, Visualization, Software, Data curation, Conceptualization. **Paul W. Bird:** Writing – review & editing, Data curation. **Christopher A. Martin:** Writing – review & editing. **Joshua Nazareth:** Writing – review & editing, Data curation. **Latif Rahman:** Writing – review & editing, Data curation. **Kate O'Kelly:** Writing – review & editing, Data curation. **Rakesh Panchal:** Writing – review & editing, Data curation. **Irfana Musa:** Writing – review & editing, Data curation. **Harshil Dhutia:** Writing – review & editing, Data curation. **Shirley Sze:** Writing – review & editing, Data curation. **Manish Pareek:** Writing – review & editing, Supervision, Formal analysis, Data curation, Conceptualization. **Michael R. Barer:** Writing – review & editing, Supervision, Formal analysis, Data curation, Conceptualization.

## Declaration of competing interest

The authors declare the following financial interests/personal relationships which may be considered as potential competing interests:

Daniel Pan is funded by a National Institute for Health and Care Research Doctoral Research Fellowship (NIHR award number: NIHR302338). The other authors declare that they have no known competing financial interests or personal relationships that could have appeared to influence the work reported in this paper.

## Appendix Supplementary materials

Image, application 1
