# Supplementary material for: Implementation of facemask sampling for the detection of infectious individuals with SARS-CoV-2 in high stakes clinical examinations – a feasibility study
Source: Future Healthc J. 2024 Sep 5;11(4):100175. doi: 10.1016/j.fhj.2024.100175 (PMC11437942; doi:10.1016/j.fhj.2024.100175)
Supplement: Supplementary file 1 [file mmc1.docx]

Appendix 1: Responses to statements from Figure 1. Number is the theme, letter is the statement, in the same order as Figure 1. For example, 1a = Mask is comfortable, 1b is Happy to wear the mask, etc.

Appendix 2: *p* values from statistical tests that analyzed responses to statements from Figure 1, by sex, grade and ethnicity. Pearson’s Chi-squared test and Fisher’s exact row test were used to compare categorical variables between groups. Student’s t-test and Kruskal-Wallis were used to compare continuous variables between groups depending on the normality of distribution.

| Question | Strongly agree (%) | Agree (%) | Neutral | Disagree | Strongly disagree |
| --- | --- | --- | --- | --- | --- |
| 1a | 8 (23.53) | 20 (58.82) | 4 (11.76) | 1 (2.94) | 1 (2.94) |
| 1b | 11 (32.35) | 19 (55.88) | 4 (11.76) | 0 | 0 |
| 1c | 11 (32.35) | 18 (52.94) | 5 (14.71) | 0 | 0 |
| 1d | 12 (35.29) | 19 (55.88) | 2 (5.88) | 0 | 1 (2.94) |
| 1e | 2 (5.88) | 5 (14.71) | 5 (14.71) | 19 (55.88) | 3 (8.82) |
| 1f | 2 (5.88) | 6 (17.65) | 2 (5.88) | 19 (55.88) | 5 (14.71) |
| 2a | 9 (26.47) | 12 (35.29) | 5 (14.71) | 8 (23.53) | 0 |
| 2b | 8 (23.53) | 18 (52.94) | 5 (14.71) | 2 (5.88) | 1 (2.94) |
| 2c | 1 (2.94) | 6 (17.65) | 9 (26.47) | 15 (44.12) | 3 (8.82) |
| 2d | 1 (2.94) | 7 (20.59) | 5 (14.71) | 17 (50) | 4 (11.76) |
| 2e | 6 (17.65) | 23 (67.65) | 4 (11.76) | 1 (2.94) | 0 |
| 3a | 0 | 2 (5.88) | 5 (14.71) | 22 (64.71) | 5 (14.71) |
| 3b | 0 | 3 (8.82) | 2 (5.88) | 22 (64.71) | 7 (20.59) |
| 3c | 0 | 3 (8.82) | 4 (11.76) | 20 (58.82) | 7 (20.59) |
| 4a | 1 (2.94) | 7 (20.59) | 5 (14.71) | 18 (52.94) | 3 (8.82) |
| 4b | 1 (2.94) | 5 (14.71) | 8 (23.53) | 17 (50) | 3 (8.82) |
| 4c | 1 (2.94) | 5 (14.71) | 6 (17.65) | 18 (52.94) | 4 (11.76) |
| 5a | 0 | 19 (55.88) | 9 (26.47) | 5 (14.71) | 1 (2.94) |
| 5b | 5 (14.71) | 25 (73.53) | 2 (5.88) | 2 (5.88) | 0 |
| 5c | 6 (17.65) | 17 (50) | 5 (14.71) | 6 (17.65) | 0 |
| 5d | 0 | 4 (11.76) | 5 (14.71) | 19 (55.88) | 6 (17.65) |
| 5e | 1 (2.94) | 23 (67.65) | 7 (20.59) | 3 (8.82) | 0 |
| 5f | 2 (5.88) | 20 (58.8) | 7 (20.59) | 4 (11.76) | 1 (2.94) |

Appendix 1.

| Question number | Sex  (p value) | Grade  (p value) | Ethnicity  (p value) |
| --- | --- | --- | --- |
| 1a | 0.42 | 0.52 | 0.24 |
| 1b | 0.52 | 0.23 | 0.58 |
| 1c | 0.81 | 0.42 | 0.75 |
| 1d | 0.27 | 0.55 | 0.41 |
| 1e | 0.61 | 0.58 | 0.50 |
| 1f | 0.28 | 0.33 | 0.07 |
| 2a | 0.10 | 0.55 | 0.20 |
| 2b | 0.28 | 0.33 | 0.74 |
| 2c | 0.72 | 0.74 | 0.12 |
| 2d | 0.79 | 0.31 | 0.76 |
| 2e | 0.86 | 0.67 | 0.18 |
| 3a | 0.61 | 0.92 | 0.20 |
| 3b | 0.84 | 1.0 | 0.71 |
| 3c | 0.70 | 0.86 | 0.84 |
| 4a | 0.28 | 0.28 | 0.17 |
| 4b | 0.15 | 0.68 | 0.70 |
| 4c | 0.50 | 0.62 | 0.21 |
| 5a | 0.53 | 0.91 | 0.88 |
| 5b | 0.70 | 0.27 | 0.49 |
| 5c | 0.68 | 0.09 | 0.24 |
| 5d | 0.62 | 0.85 | 0.57 |
| 5e | 0.43 | 0.87 | 0.89 |
| 5f | 0.73 | 0.75 | 0.49 |

Appendix 2
